# Analysis of the codon usage pattern in Middle East Respiratory Syndrome Coronavirus

**DOI:** 10.18632/oncotarget.22738

**Published:** 2017-11-27

**Authors:** Ye Chen, Quanming Xu, Xiaomin Yuan, Xinxin Li, Ting Zhu, Yanmei Ma, Ji-Long Chen

**Affiliations:** ^1^ Key Laboratory of Fujian-Taiwan Animal Pathogen Biology, College of Animal Sciences, Fujian Agriculture and Forestry University, Fuzhou, 350002, China; ^2^ College of Life Sciences, Fujian Agriculture and Forestry University, Fuzhou, 350002, China; ^3^ CAS Key Laboratory of Pathogenic Microbiology and Immunology, Institute of Microbiology, Chinese Academy of Sciences, Beijing 100101, China

**Keywords:** MERS-CoV, codon usage pattern, mutation bias, natural selection

## Abstract

Middle East Respiratory Syndrome Coronavirus (MERS-CoV), which first broken out in Jeddah in 2012, causes a severe acute respiratory illness with a high mortality rate. To better understand the molecular characteristics of isolated MERS-CoV genomes, we first analysed the codon usage pattern of the zoonotic MERS-CoV strains comprehensively to gain an insight into the mechanism of cross-species transmission. We found that MERS human/camel isolates showed a low codon usage bias. Both mutation and nature selection pressure have contributed to this low codon usage bias, with the former being the main determining factor. We also observed that gene function, evolution time and the different host species of the virus all contributed to the bias of MERS-CoV, to some extent. Additionally, the codon usage pattern of MERS-CoV isolates is different from other related Nidovirales viruses isolated from bats and hedgehogs. In the future, more epidemiological surveys are required to examine the factors that resulted in the emergence and outbreak of this virus.

## INTRODUCTION

Coronavirus (CoV), a positive sense, single-stranded RNA virus, was first reported in 1949 [1]. It belongs to the family Coronaviridae and ranges from 26 to 32 kb in length, making it the largest known RNA virus [2]. To date, six CoVs have been identified that infect humans, including Human CoV-229E (HCoV-229E), Human CoV-OC43 (HCoV-OC43), Severe Acute Respiratory Syndrome CoV (SARS-CoV), Human CoV-NL63 (HCoV-NL63), Human CoV- HKU1 (HCoV-HKU1) and Middle East Respiratory Syndrome (MERS-CoV) [3]. To date, WHO has reported 2081 laboratory-confirmed cases infected with MERS-CoV, including 722 deaths (http://www.who.int/emergencies/mers-CoV/en/), making MERS-CoV one of the most dangerous viruses known to humans. Previous studies indicated that MERS-CoV might have recombination events in different lineages [4]. Previous study also showed that the MERS-CoV species and HCoV-229E-related lineage co-circulated in Saudi Arabia, and they found a recombinant lineage of MERS-CoV that is endemic in camels [5]. The recombinant viruses led to an outbreak of MERS-CoV in humans in 2015 [6]. The evolution rate and recombination rate of coronavirus is increasing, such as MERS-CoV [4], therefore, it's significantly important to study the evolution and the influencing factors of MERS-CoV.

Codon usage bias is an important indicator of genome evolution. There are several factors that might influence the codon usage bias, including natural selection, mutational pressure, G+C content, secondary protein structure and replication selective transcription [7, 8]. Codon usage is a driving force in the evolution of small DNA viruses and astroviruses [9]. Some studies showed that the codon usage bias of RNA viruses is low, such as in the Equine infectious anemia virus (EIAV) [10], Zaire ebolavirus (ZEBOV) [11], the N gene of Rabies virus (RABV) [12] and Porcine epidemic diarrhea virus (PEDV) [13]. A previous study indicated that different SARS-CoV genes had significant variation in their codon usage bias [14]; however, the bias is low. In contrast, Woo et al. demonstrated that CoV-HKU1 has a strong codon usage bias and a high NNU/NNC ratio of 8.835 [15]. They also showed that both cytosine deamination and selection of CpG-suppressed clones are the major factors that shape codon bias in CoV genomes [15]. Additionally, a previous study showed that the codon usage of HCoV-NL63 is characterised by a high U composition and a low G/C composition, which might reflect the evolutionary origin of the virus. They suggested that viruses acquire some functions from other recent viral or cellular origins by gene transfer [16]. During protein biosynthesis, synonymous codon encoded amino acids are not used randomly, and some species or genes always prefer to use of one or several particular synonymous codons, which was termed as codon usage bias. Previous studies revealed that different genes from different species or from the same species have obvious codon usage biases [14, 17]. In the present study, we first analysed the codon usage data of MERS-CoV strains. The codon usage information for the MERS-CoV strains might provide some clues to the characteristics of the MERS genome and the evolutionary history of the virus.

## RESULTS

### Composition of MERS-CoV and the related CoV isolates

The compositions of the 32 human related MERS-CoV, the 24 camel related MERS-CoV, the 12 bat related MERS-CoV and the 3 hedgehog related MERS- CoV were analysed and shown in Table 1. The results showed that all of the MERS-CoV strains and MERS related strains were poor in C/G and rich in A/U.

**Table 1:** The nucleotide contents of MERS and MERS related isolates

Accession	Nucleotide content				Accession	Nucleotide content			
	A%	C%	G%	T%		A%	C%	G%	T%
KC667074.1	26.13	20.15	21.25	32.47	KT368826.1	26.15	20.14	21.23	32.48
KC164505.2	26.12	20.10	21.23	32.54	KT751244.1	26.13	20.11	21.24	32.52
KF600612.1	26.13	20.14	21.29	32.44	KP719931.1	26.14	20.12	21.24	32.50
KF600652.1	26.13	20.16	21.25	32.46	KJ650098.1	26.14	20.14	21.24	32.48
KF600620.1	26.13	20.13	21.29	32.44	KM027260.1	26.30	19.92	21.21	32.56
KM210277.1	26.14	20.13	21.25	32.48	KM027256.1	26.30	19.92	21.21	32.56
KM210278.1	26.14	20.14	21.25	32.47	KT368857.1	26.15	20.10	21.24	32.51
KM015348.1	26.14	20.14	21.25	32.48	KT368852.1	26.15	20.10	21.24	32.50
KF600651.1	26.13	20.16	21.24	32.47	KT368837.1	26.16	20.12	21.22	32.50
KF600647.1	26.13	20.16	21.24	32.47	KT368824.1	26.16	20.12	21.23	32.49
KF600627.1	26.13	20.16	21.24	32.47	KT368890.1	26.15	20.11	21.23	32.50
KF600630.1	26.13	20.15	21.26	32.46	KT368885.1	26.15	20.12	21.23	32.49
KJ156952.1	26.13	20.15	21.26	32.45	KT368881.1	26.16	20.12	21.23	32.50
KJ156949.1	26.13	20.11	21.26	32.50	KT368879.1	26.15	20.12	21.24	32.49
KJ156869.1	26.13	20.15	21.25	32.48	KT368873.1	26.15	20.12	21.24	32.49
KJ156866.1	26.13	20.16	21.24	32.47	KT368877.1	26.14	20.12	21.25	32.49
KP209312.1	26.12	20.09	21.23	32.56	KT368867.1	26.15	20.09	21.25	32.51
KJ813439.1	26.12	20.08	21.24	32.55	KT368860.1	26.17	20.11	21.21	32.51
KT121581.1	26.19	20.27	20.96	32.58	KT368858.1	26.17	20.11	21.21	32.51
KT121580.1	26.19	20.26	20.96	32.59	KT368859.1	26.17	20.11	21.21	32.51
KT121573.1	26.19	20.26	20.96	32.59	EF065509	26.50	21.55	21.71	30.24
KT121575.1	26.19	20.26	20.96	32.59	EF065510	26.57	21.44	21.62	30.37
KP209313.1	26.11	20.10	21.24	32.55	EF065511	26.88	21.44	21.62	30.36
KP209311.1	26.11	20.10	21.24	32.55	EF065512	26.58	21.44	21.62	30.36
KP209310.1	26.11	20.12	21.23	32.54	NC009020	26.50	21.55	21.71	30.24
KT006149.2	26.14	20.11	21.24	32.51	EF065505	27.54	17.16	20.72	34.58
KT225476.2	26.14	20.15	21.22	32.49	EF065506	27.54	17.16	20.73	34.57
KT029139.1	26.12	20.08	21.22	32.58	EF065507	27.54	17.16	20.73	34.58
KU308549.1	26.14	20.11	21.25	32.50	EF065508	27.53	17.14	20.75	34.58
KT026456.1	26.12	20.09	21.23	32.56	KJ473822	27.56	17.06	20.83	34.54
KT374054.1	26.14	20.11	21.25	32.50	KC869678.4	26.45	19.05	21.28	33.21
KT374050.1	26.15	20.11	21.24	32.51	KJ473821.1	25.14	20.77	22.55	31.53
KF961222.1	26.21	20.31	20.99	32.49	NC-022643.1	29.17	16.16	21.43	33.24
KF961221.1	26.19	20.24	20.96	32.59	KC545386.1	29.17	16.16	21.43	33.24
KJ477102.1	26.09	20.11	21.23	32.58	KC545383.1	29.16	16.13	21.43	33.28
KJ713299.1	26.13	20.10	21.25	32.51					

The accession numbers marked in red, green, blue and black represent the MERS-CoV human isolates, the MERS-CoV camel isolates, the MERS-CoV related CoV bat isolates and the MERS-CoV related CoV hedgehog isolates, respectively.

The most and least abundant bases were U and C, respectively. The SD value, calculated for different kinds of isolates based on the nucleotide abundance (Supplementary Table 2), showed that the value of A and U were small in the four nucleotides in the MERS-CoV human/camel isolates, although it was lowest in the CoV hedgehog isolates and largest in the CoV bat isolates, respectively. This finding suggested that the base contents vary non-significantly between the MERS-CoV isolates in human/camel strains, and hedgehog related CoV. However, the CoV isolates from bat showed a larger amount of variation.

### Synonymous codon usage in MERS human isolates

**Figure 1:**
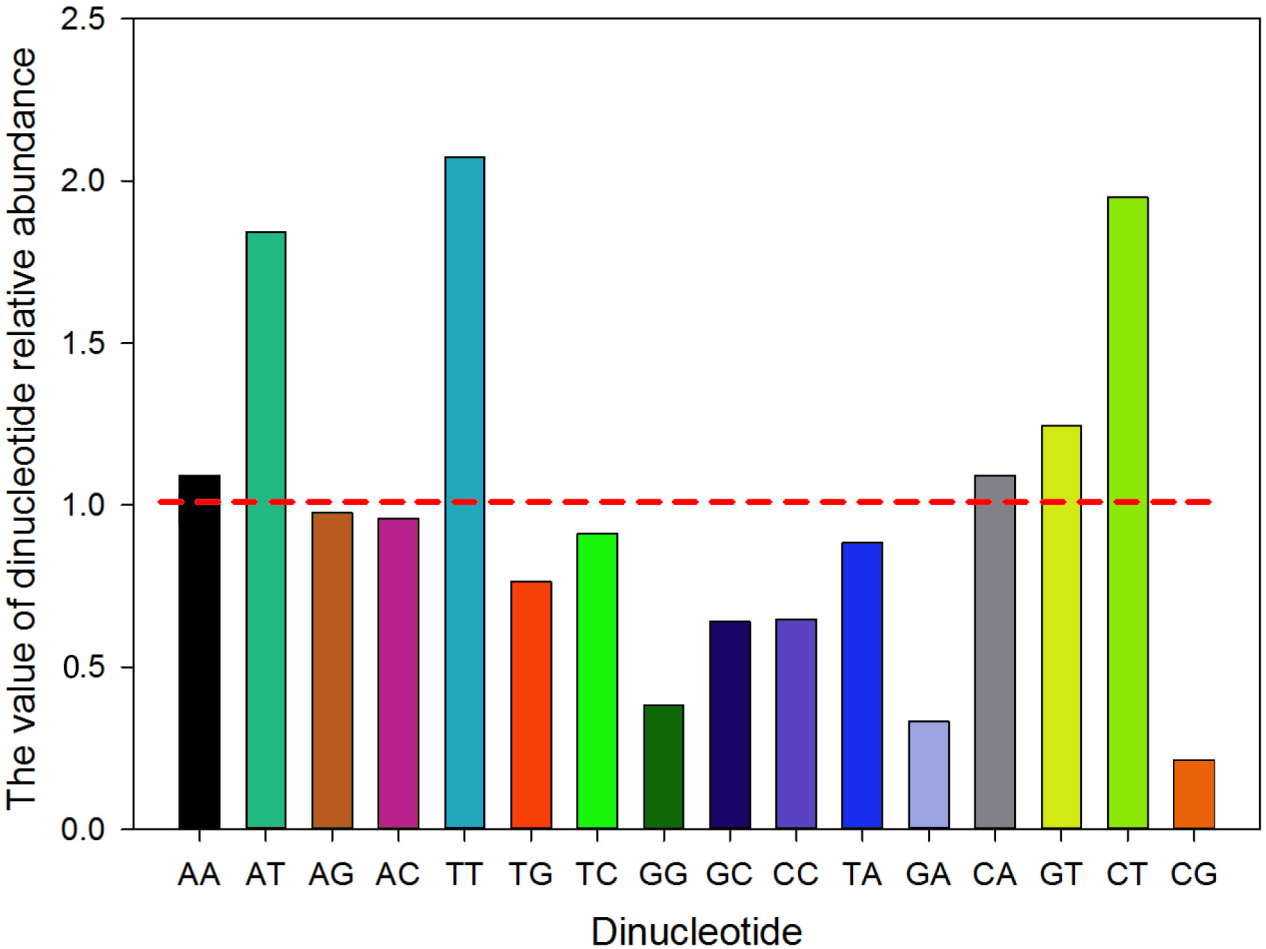
The relative abundance values of the 16 dinucleotides The different colours represent the different dinucleotides. The red dotted line indicates that the relative abundance value of a dinucleotide is 1.

The relative synonymous codon usage (RSCU) value of each synonymous codon in the MERS-CoV genome was calculated (Table 2), which indicated that codon usage bias exists in the coding sequence of the MERS-CoV genome. Among codons encoding hydrophobic amino acids, CCG (proline, 868 times) and GUU (valine, 17588 times) were the least and most frequently used codons, respectively. Meanwhile, among the hydrophilic amino acids, the least and most frequently used codons were CGG (arginine, 1160 times) and GAU (aspartic acid, 14947 times), respectively. We also observed that there were no synonymous codons encoding an amino acid with the same RSCU value, which indicated that synonymous codons are not used equally in MERS-CoV human isolates. In addition, the 18 most frequently used codons for each amino acid ended in either U or A. Meanwhile, among the synonymous codons of the 18 amino acids, 15 codons ended with U and 3 ended with A, which further proved that the codon bias exists in the MERS-CoV human isolates. To estimate the degree of codon usage bias in the MERS-CoV human isolates genome, the effective number of codons (ENC) value of the 32 strains were calculated. The average ENC value was 49.816 ± 0.08 which was high (>45) and indicated a lower codon usage bias existed in MERS-CoV. The relative abundance values of the 16 dinucleotides were then counted (Figure 1). We noted that most of the relative abundance values of the 16 dinucleotides were not in accordance with expected value (i.e. the relative abundance value=1). And 10 of the dinucleotides (AG, AC, UG, UC, GG, GC, CC, UA, GA and CG) have less than 1 relative abundance values, however, the other six dinucleotides (AA, AU, UU, CA, GU and CU) were larger than 1. This indicated that the codon usage pattern is not equal for these dinucleotides. Similarly, CpG had the lowest frequency, while UpU showed the highest frequency. Hence, this analysis suggested that the composition of the nucleic acids affected the codon usage pattern of MERS-COV.

**Table 2:** The synonymous codon usage pattern presented in the MERS strains

AA	Codon	RSCU/number	AA	Codon	RSCU/number
A (Ala)	GCA	0.988/7894	P (Pro)	CCA	1.216/5631
	GCC	0.632/5061		CCC	0.656/3032
	GCG	0.308/2476		CCG	0.188/868
	***GCU***	***2.068/16549***		***CCU***	***1.94/8980***
C (Cys)	UGC	0.806/5335	Q (Glu)	***CAA***	***1.14/8767***
	***UGU***	***1.194/7910***		CAG	0.86/6620
D (Asp)	GAC	0.72/8395	R (Arg)	***AGA***	***1.344/3580***
	***GAU***	***1.28/14947***		AGG	0.84/2242
E (Glu)	***GAA***	***1.05/8926***		CGA	0.456/1210
	GAG	0.95/8073		CGC	1.104/2948
F (Phe)	UUC	0.718/8016		CGG	0.432/1160
	***UUU***	***1.282/14285***		***CGU***	***1.824/4872***
G (Gly)	GGA	0.644/4116	S (Ser)	AGC	0.438/2457
	GGC	1.008/6467		AGU	1.332/7527
	GGG	0.292/1883		UCA	1.212/6838
	***GGU***	***2.052/13156***		UCC	0.714/4035
H (His)	CAC	0.682/3078		UCG	0.186/1065
	***CAU***	***1.318/5955***		***UCU***	***2.112/11933***
I (Ile)	AUA	0.714/5048	T (Thr)	ACA	1.180/8958
	AUC	0.573/4060		ACC	0.688/5243
	***AUU***	***1.713/12116***		ACG	0.176/1336
K (Lys)	***AAA***	***1.004/11594***		***ACU***	***1.956/14865***
	AAG	0.996/11513	V (Val)	GUA	0.724/7162
L (Leu)	CUA	0.456/3141		GUC	0.764/7536
	CUC	0.708/4859		GUG	0.736/7262
	CUG	0.486/3359		***GUU***	***1.78/17588***
	***CUU***	***1.698/11693***		TAC	0.732/7604
	UUA	1.218/8373		***UAU***	***1.268/13190***
	UUG	1.434/9867		***UAU***	***1.268/13190***
N (Asn)	AAC	0.604/6647			
	***AAU***	***1.396/15362***			

The bold and italic text indicates the preferentially used codons and RSCU values for the MERS strains.

The preferentially used codons for each amino acid are displayed in bold and italics.

### Mutational bias influences the codon usage bias of MERS-CoV and MERS related strains

**Figure 2:**
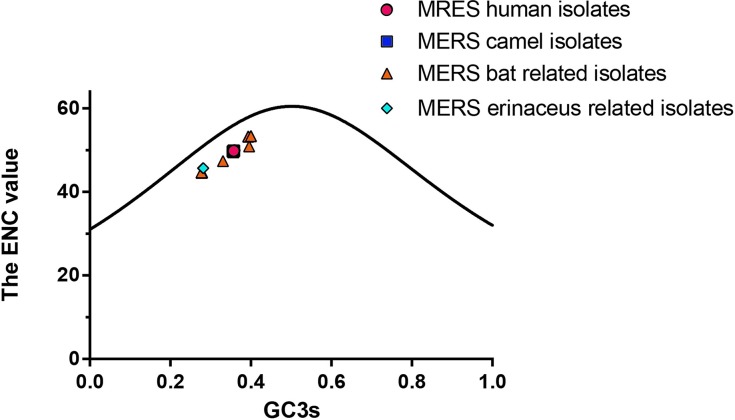
The plots of ENC values against GC3s values for MERS-CoV and MERS-CoV related strains All the points corresponding to human, camel isolated MERS-CoV strains and bat and hedgehog(erinaceus) isolated CoV were labelled in circle, square, triangle, and rhombus, respectively.

To further investigate which factors account for the low codon usage bias of MERS-CoV and the related viruses, we analysed the relationship between the ENC value and the percentage of G or C in the third site of codons (GC3s) % in MERS-CoV genomes. In Figure 2, the solid line represents the curve produced if the codon usage is only determined by the GC3s [18]. A large proportion of points lying near to the solid line on the left region of this distribution would suggest that mutational bias is the main factor determining the codon usage variation among these genes.

The research object was divided into four parts: MERS-CoV human isolates, MERS-CoV camel isolates, bat related MERS-CoV strains and hedgehog related MERS-CoV strains. The ENC values of the coding regions of these strains were plotted against the GC3s (Figure 2). It's shown that the observed value was smaller than the expected value, which indicated that the codon usage bias patterns exist in these MERS-CoV strains. From the resultant Figure 2, the results showed that the ENC values of the 32 MERS-CoV human isolates and 24 MERS-CoV camel isolates were clustered together, with few changes between each other. This indicated that the ENC value of MERS-CoV human isolates or camel isolates changed little between different strains, which was in accordance with the small ENC SD value of these sequences. We also observed that the ENC values of bat and hedgehog CoV isolates show slightly higher dispersion levels compared with each other. However, most of the plots for bat CoV strains surrounded the MERS-CoV human and camel isolates and were not far from them; the plots for the hedgehog isolates were a little further from them. This analysis showed that the mutation pressure affects the codon usage bias of these strains. Besides mutational bias, there might be additional factors that drive the codon usage variation among these genes. It is generally recognized that factors such as mutational bias and natural selection pressure contribute to codon usage bias patterns [19–21]. Thus, to further investigate the possible influence of mutational pressure on the MERS-CoV strains codon usage bias patterns, correlation analysis was performed between the codon compositions (A3s, U3s, G3s, C3s, and GC3s), the ENC values and nucleotide compositions (A%, U%, G%, C%, and GC%) (Table 3). The results revealed that most of the codon compositions correlated with the nucleotide compositions. Among them, the U3s, A3s, G3s, GC3s correlated significantly with almost all the nucleotide compositions (A%, U%, G%, C%, and GC%), with P values less than 0.01. These results confirmed that the codon usage bias of the MERS-CoV strains (human/camel isolates) was influenced by the nucleotide compositions; thus by mutational bias.

**Table 3:** The correlations between the codon compositions (A3s, U3s, G3s, C3s, and GC3s), the ENC values, nucleotide compositions (A%, U%, G%, C%, and GC%), the first axis values, the second axis values, the Gravy values, and the Aroma values of the MERS human isolates

	A%	C%	G%	U%	GC%	1st axis	2nd axis	Gravy	Aroma
U3s	0.687^**^	0.665^**^	-0.913^**^	0.904^**^	-0.831^**^	0.924 ^**^	-0.224	-0.882^**^	0.983^**^
C3s	0.012	-0.177	0.172	-0.185	0.240	-0.209	0.091	0.218	-0.251
A3s	-0.276	-0.406	0.634 ^**^	-0.803^**^	0.775 ^**^	-0.670^**^	0.436*	0.636 ^**^	-0.790^**^
G3s	-0.772 ^**^	-0.784 ^**^	0.959^**^	-0.819^**^	0.718^**^	-0.958 ^**^	0.050	0.933^**^	-0.966^**^
ENC	0.002	0.067	0.337	-0.895^**^	0.736^**^	-0.359	0.812 ^**^	0.270	-0.576 ^**^
GC3s	-0.716 ^**^	-0.620 ^**^	0.898^**^	-0.920^**^	0.817 ^**^	-0.886^**^	0.232	0.835^**^	-0.945 ^**^

^*^ Signifies 0.01 <P-value < 0.05 and ^**^ signifies a P-value < 0.01, indicating significant and highly significant correlations, respectively.

We observed that the data points of MERS-CoV human and camel isolates were clustered around the origin and did not diverge too much from each other, while the related bat and hedgehog CoV isolates were dispersed and diverged from each other; they did not cluster around the origin, and were close to either 1st or 2nd axis. We then performed correlation analysis between the codon compositions, and the first axis value and the second axis value revealed these compositions were correlated or significantly correlated (Table 3), especially for the first axis. These observations reflected that: (1) there was little change in the codon usage bias between the MERS-CoV human isolates and the MERS-CoV camel isolates. This also proved that mutational bias contributed to the MERS-CoV codon usage bias. This was also in accordance with the SD values of the RSCU of the MERS human/camel isolates; (2) In addition to mutational pressure, there are other factors, such as natural selection, which might influence the codon bias of MERS-CoV of human/camel isolates; (3) CoV of *Tylonyteris*, *Pipistrellus* and *Erinaceus europaeus* have distinct codon usage patterns compared with the MERS-CoV human/ camel isolates: in the plots, the data points were closer to the axes than to the origin; (4) The codon usage bias patterns of *Tylonyteris*, *Pipistrellus* and *Erinaceus europaeus* CoV were not only caused by mutational bias, but also by natural selection pressure, which might have had a larger effect than mutational bias.

### Natural selection influences the codon usage bias of MERS human isolates

Natural selection plays an important role in codon usage bias of MERS-CoV and its related strains. To investigate the effect of natural selection pressure on the MERS-CoV codon usage bias, correlation analysis was studied between the Gravy and Aroma values and the codon compositions (Table 3). The results indicated that the Gravy value was significantly correlated with the A3s, U3s, G3s and GC3s and that the Aroma value was correlated significantly with the A3s, U3s, G3s, GC3s, and the ENC value, further confirming that natural selection influenced the MERS-CoV strains’ codon usage bias. The codon usage patterns of *Homo sapiens*, *Camelus dromedaries*, *Taphozous*, *Pipistrellus pipistrellus*, *Erinaceus europaeus* are available online (http://www.kazusa.or.jp/codon/). We obtained the RSCU values of the hosts of these coronavirus strains online (Supplementary Table 3). It's revealed that among most of the preferred synonymous codons in the human/camel hosts and viral genomes of MERS-CoV strains, there were no same codon bias. However, we also observed that for 10 codons encoding each amino acid (C, D, E, F, H, I, K, N, Q, R) in *Pipistrellus pipistrellus*, their preferred codons were the same as those in the MERS-CoV human/camel isolates. 5 codons encoding each amino acid (D, I, K, N, P) in *Taphozous* used the same codons and 3 codons encoding each amino acid (A, E, P) in *Erinaceus europaeus* used the same preferred codons as the MERS human/camel isolates.

### Does mutational bias or natural selection pressure have a decisive role in forming the MERS human/camel isolates codon usage patterns?

**Figure 3:**
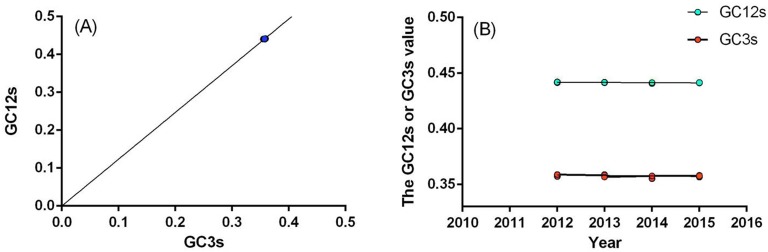
**(A)** The neutral analysis of GC3s against GC12s. **(B)** The evolutionary analysis of the GC3s and GC12s values. The solid line represents the regression line.

To distinguish the roles of mutational bias and natural selection in shaping the MERS codon usage patterns, the GC12s (the mean value of GC1s and GC2s) was plotted against the GC3s (Figure 3A). In the analysis, the GC12s was correlated with the GC3s (r = 0.9525, P< 0.0001). The correlation coefficient was 0.953 ± 4.840951e-017, which indicated that the relative neutrality was 95.3%, in other words, the relative constraint of GC3s was 4.7%, which demonstrated that mutational bias had a much larger influence than natural selection pressure on the MERS-CoV of human/camel isolates coding sequences.

### Evolutionary analysis of codon usage patterns

To uncover the evolutionary pattern of the MERS human/camel isolates’ codon usage biases, the GC12s and GC3s were plotted against evolutionary time from 2012 to 2015, respectively (Figure 3B). Both the GC12s and the GC3s values were negatively correlated with time (for GC12s, r =-0.5736 and P < 0.0001; for GC3s, r =-0.4389 and P = 0.0120). The change rete of the GC12s and GC3s were -0.0001918 and 0.00017765 bases per year, respectively. The data suggested that the GC content at all three codon positions decreased as the evolution of MERS-CoV human/camel isolates. Additionally, the evolution rate of the GC3s was very similar to that of the GC12s, indicating that compared with the natural selection pressure, the mutational bias played an increasingly important role in shaping the MERS human/camel codon usage pattern.

**Figure 4:**
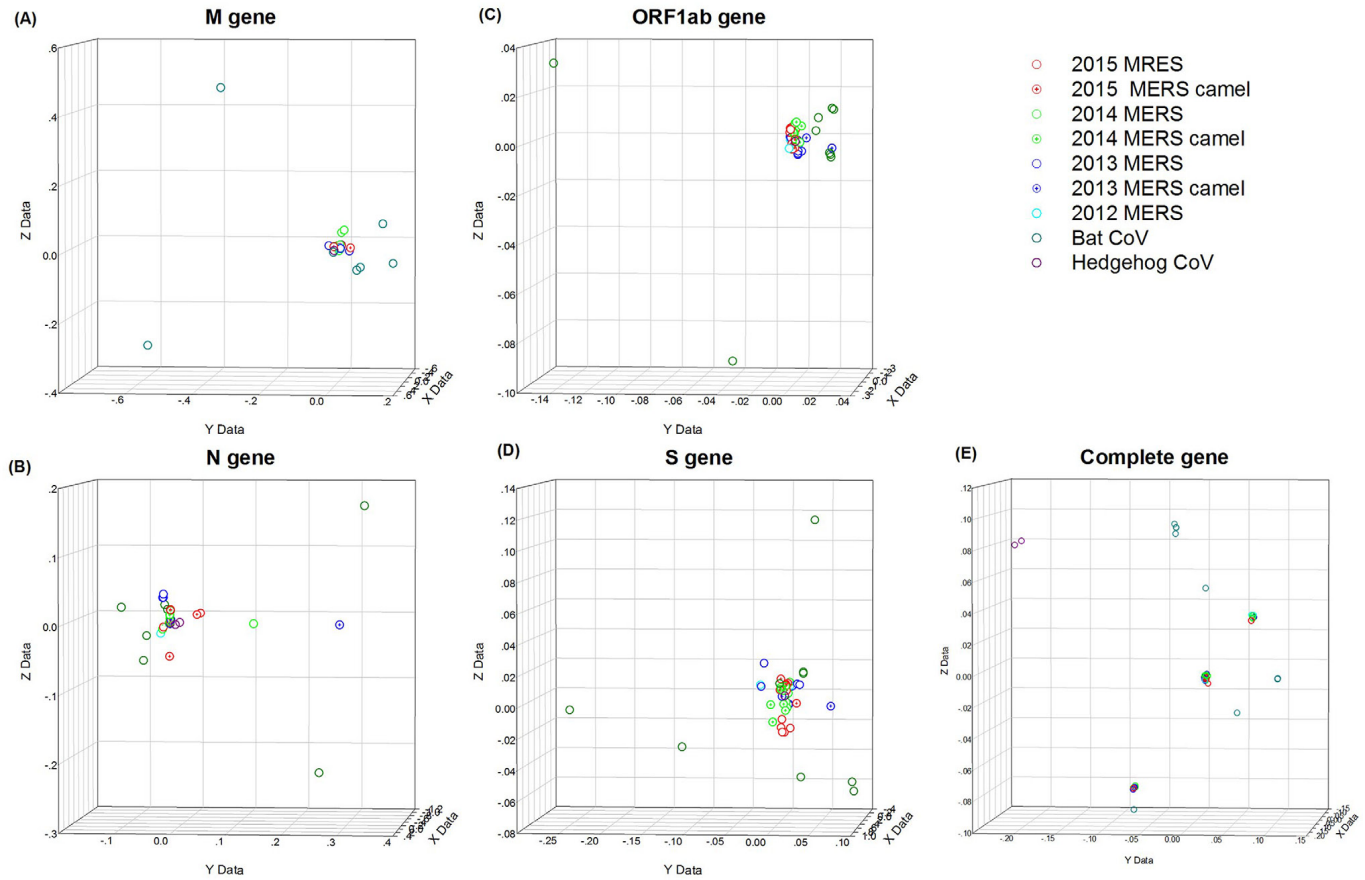
CA of MERS-CoV human/camel isolates Each viral gene is displayed in a 3-dimensional representation. The X, Y and Z-axes have arbitrary scales generated by the CA and the weight of each codon in these axes varies in different segments. The codon usage trends with time of the viral isolates are indicated by different colours. The different hosts of the MRES-CoV isolates are indicated by different shapes. **(A)**, **(B)**, **(C)**, **(D)** and **(E)** represents the 3D graph of the M, N, ORF1ab, S and the complete genome, respectively, using the CA data.

Then the possible connection between the codon usage pattern and the evolution of sequences/genes were further investigated. Here, the first three axes from the correspondence analysis (CA) were used to provide a 3-dimensional visualization of the relationships among the sequences. Colours were used to identify sequences with different features (e.g. viral host and year of isolation) in the analysis. The phylogenetic tree analysis which performed by the previous report method found that these 54 MERS-CoVs were divided into two clades, named Clade A and Clade B (Supplementary Figure 1), and the results are consistent with previous study [4]. In this analysis, the different open reading frames (ORFs) of the whole genome and different genes of MERS-CoV strains were used (Figure 4). For the M gene, the gene changes seemed to be random, which indicated that evolution had little effect on the M gene (Figure 4A). For the ORF1ab gene, there was a clear evolutionary trend over time, especially for the 2015 isolates of MERS-CoV (Figure 4C). For the N gene, mutation was the main influencing factor (Figure 4B). The result for the S gene was similar to the ORF1ab gene (Figure 4D): both evolution and mutation affected the codon usage pattern, with mutation having the larger effect. Obviously, the aggregation/dispersion states of the plots that were composed using different genes are different to the plots using the whole genome in Figure 4E. Thus, the different genes contributed to the MERS-CoV codon usage bias by different degrees.

## DISCUSSION

The genetic code is degenerate, as multiple codons code for a single amino acid. Most organisms exhibit differences in base composition and significant codon bias (unequal usage of synonymous codons). Generally, mutations leading to change in amino acids are studied as a measure of selection. Synonymous mutations can change the base composition of genes without altering the corresponding proteins. Intuitively, synonymous mutations appear to be “neutral” or “near-neutral” in their effects; however, their evolutionary consequences are being recognised increasingly [22–26]. Studies show that codon bias and synonymous mutations are under weak selection, driving evolution in various organisms [27–29]. Genes that are enriched for preferred codons are known to have higher translational efficiency. It has been shown in other host-pathogen systems, such as bacteria–bacteriophages, that long-term co-evolution has resulted in some genes of bacteriophages being enriched in the codons preferred by their respective bacterial hosts [30]. A balance between selection, mutation and genetic drift maintains the codon bias in the host and the pathogens. Thus, studies revealing determinants of the bias and its dynamics are central to our understanding of host–pathogen evolution [31, 32]. Previous studies has been revealed that compared with DNA viruses, the evolution of RNA viruses was faster, such as the evolution of influenza virus [33, 34], coronaviruses [4, 35]. Codon usage analysis is a serviceable and well-established method to study the codon usage patterns of different organisms, such as the studies of codon usage of VP2 gene of canine parvovirus [36], and the N gene of rabies viruses [12]. Recently, the high case–fatality ratio of MERS-CoV infection has attracted considerable attention in the worldwide. Although the genome sequence of MERS-CoV has been published and many studies have been performed after each outbreak [4, 6, 37, 38], little genomic analysis was studied on this virus. To further understand the genomics of the MERS strains, we performed codon usage analysis of the MERS-CoV and its related strains. Investigating the extent and causes of codon usage bias is essential for research focused on viral evolution and transmission. To investigate the factors leading to the MERS-CoV and MERS related strains codon usage patterns, several analytical methods were used in our study. First, the RSCU value of the MERS strains were calculated. The results indicated that codon usage bias exists and that the MERS preferred codons almost all end in U, with a proportion of 15/18. The codon usage bias was further confirmed by the mean ENC value of 49.82. For comparison, the mean ENC value for other studied viruses were: Porcine epidemic diarrhea virus (mean ENC=47.91) [13], SARS (mean ENC = 48.99)[39], Foot and mouth disease virus (mean ENC = 51.42) [40], H5N1 influenza A virus (mean ENC = 50.91) [41], Duck enteritis virus (mean ENC = 52.17) [42], Classical swine fever virus (mean ENC = 51.7) [43] and Hepatitis A virus (mean ENC = 39.78)[44]. An ENC value greater than 45 is considered as a lower codon usage bias. The mean ENC value for MERS-CoV strains was a little higher than most viruses and was higher than 45; therefore, the codon usage bias of MERS is relatively low. The codon studies on coronavirus has been reported previously [39, 45], however, the ENC of MERS-CoV was higher than the SARS CoV, additionally, in this study, we discovered that other than mutation pressure, natural selection, as well as the abundance of dinucleotide, also contribute to the evolution of MERS-CoV. A low biased codon usage pattern might allow the virus make use of several codons for each amino acid, and might be beneficial for viral replication and translation in the host cells. The relative abundance of dinucleotides also correlated with the first two principal components in the PCA analysis. The result showed that there was an obvious distinction in the dinucleotide usage. CpG dinucleotides had the lowest abundance, which indicated that MERS-CoV might have the ability adapt to the host. When the ENC values were plotted against GC3s, the codon usage bias in MERS-CoV was identified. If the codons of MERS-CoV strains were completely random, with bias supplied in the standard curve, all of the data points would lie upon the expected curve. However, Figure 2 showed that the data points representing the ENC value for each MERS-CoV strain were lower than the expected curve. This indicated that there were codon usage bias in the codon usage pattern, and other factors influenced the MERS codon usage pattern. Additionally, in the ENC-plot analysis, it was discovered that bat CoV isolates show slightly higher dispersion levels, and near to the distribution of human related and camel related CoVs, which might due to the consequence of the bat was the natural host of coronavirus. Generally, the main causes of codon usage bias are considered to be mutational bias and natural selection pressure, which are the two main forces involved in shaping the synonymous codon usage pattern of RNA viruses. To confirm the possible role of mutational bias in the codon usage pattern, we performed correlation analysis between the nucleotide content and the codon composition. The strong correlation between these two variables (except in the C3s and A%, U%, G%, C%) showed that mutational bias contributed to the codon usage pattern. A significant correlation was shown between the GC3s values and the nucleotide content (Table 3), which also revealed the importance of mutational bias. The role of mutational bias was further demonstrated by the PCA analysis, which showed that the first and second components were significantly correlated with the nucleotide content. For the MERS human/camel isolates, a weak codon usage bias might be caused by natural selection when the viruses adapt to the host cells. In contrast, a strong bias caused by nature selection in the other CoV strains of MERS related isolates were also observed. With natural selection pressure, the rate of codon change might be slightly larger compared with the data whose plots are clustered close to the origin. To further determine the roles of mutation, natural selection and evolution in the MERS-CoV strains, CA was performed. CA indicated that both mutation and natural selection affect the codon usage pattern, with mutation having a more important role. This implied that there is some correlation between the different isolates. Next, we investigated the role of natural selection in shaping the MERS-CoV strains codon usage patterns by investigating the relationships between the Gravy value, the Aroma value and nucleotide content, and the high codon adaptation index (CAI) value compared with the host genome codon usage pattern. The results showed that mutation bias was more important than natural selection pressure in neutral analysis in MERS-CoV human/camel isolates, which agreed with the result of the PCA. In addition, gene function, the evolution factor and the different hosts were also identified as factors that are influential in shaping the MERS-CoV codon usage pattern, while the geographical distribution had no influence on the MERS-CoV codon usage bias.

In summary, our study identified that variation in the MERS-CoV codon usage pattern is low. Two main factors, mutational bias and natural selection pressure, have contributed to the codon usage pattern, with the former having a larger effect in MERS-CoV human/camel isolates, and the latter playing a more critical role in the CoV strains of bat/hedgehog isolates. There was a significant variation in codon usage bias between MERS-CoV human/camel isolates and the CoVs isolated from bats and hedgehogs.

The codon usage bias of MERS-CoV was different in the isolates of the latter two hosts, in which nature selection pressure played an important role in the codon usage bias. We also observed a difference between the human MERS-CoV isolates and the bat/hedgehog isolates in their use of the most preferred synonymous codon. However, among the CoV isolates from bats or hedgehogs, the use of the same synonymous codons as their hosts was highly consistent. This may hint that coronavirus does not spread so widely in humans. In the evolutionary process, natural selection pressure plays an increasing role. In addition, other factors, such as gene function, and the different outbreak times also influenced the codon usage bias to some extent. However, the geographical distribution did not have a significant role in the MERS-CoV codon usage bias.

In conclusion, this first systemic analysis of the codon usage patterns of MERS-CoV strains and the related strains will be beneficial to further studies examining this important zoonotic pathogen.

## MATERIALS AND METHODS

### Selection of sequence data

The complete genomes of different MERS and MERS related isolates were retrieved from GenBank (http://www.ncbi.nlm.nih.gov/nuccore/?term=Middle+East+respiratory+syndrome+coronavirus%3B+complete+genome, and http://www.ncbi.nlm.nih.gov). Then the sequences were selected according to their geographical distribution, the isolation date, and the host species [46–50]. To analyse the codon usage bias of MERS strains, we selected only those viruses with complete genome and complete CDS information. Detailed information about the 71 MERS and MERS related strains, including their accession number, the date they were isolated, and their place of isolation listed in the supplementary materials (Supplementary Table 1). The edited data were then aligned using the MEGA7, the BioEdit (version 7.0.9.0) sequence analysis program and the Clustal W method.

For all selected and analysed sequences, short (<300bp of the corresponding gene) and abnormal sequences were removed from the datasets, and only six viral genes were studied because the short length and insufficient codon usage diversity of the other genes might have biased the results. The six genes analysed were the E, M, N, S, ORF1ab and ORF3, and all these genes were classified according to their viral isolation date and location.

### Nucleotide composition

The nucleotide content (A%, U%, G% and C%) of each MERS and MERS related strain was analysed using BioEdit. The nucleotide composition of the synonymous codon position of each codon (GC1s%, GC2s%, GC3s%) was calculated using the cusp program online (http://emboss.toulouse.inra.fr/cgi-bin/emboss/cusp). The A3s, U3s, G3s, GC% and GC3s were calculated using the Codon W program online (http://mobyle.pasteur.fr/cgi-bin/portal.py?#forms::CodonW).

### Codon usage indices

The RSCU values were first proposed in 1986 [51] to standardize the codon usage of those amino acid encoded by different codons. The RSCU value is independent of the amino acid composition and has been used widely to estimate the codon usage bias among genes. A higher RSCU value means that the codon is used more frequently or has a higher codon usage bias. If the RSCU value of a specific codon is higher than 1.0, it is considered to be a positive codon usage bias. While the RSCU value is less than 1.0, it is considered to be a negative codon usage bias.

The ENC value is not influenced by the amino acid composition or the gene length. In the ENC analysis, an ENC value is given to each codon. The ENC value ranges from 20 to 61. In contrast to the RSCU value, a higher ENC value correlates to a weaker codon usage bias. If the codon usage of one gene is completely random and unbiased, then the expected ENC value is calculated from the GC3s [18]:

$$\text{ENC}=2+s+29/(s^{2}+{\left(1-s\right)}^{2}),$$

where the s value is the GC3s content of each codon. When the expected ENC value is plotted against the GC3s value, an expected curve is formed. A dot located on the curve is regarded as unbiased.

The relative abundance of dinucleotides were also correlated with the first two principal axes. There are two explanations for the low frequency of CpGs. The first one is that cytosine (C) is the methylation signal and the methylation of C results in a decrease in the level transcription and an increase of the mutation frequency. Thus, for codons such as XCA, XCT/U, XCC and XCG, which encode the same amino acid, the nucleotide C with an A, U or C tag after is more favourable than G. The specific oligodeoxynucleotide of the core unmethylated CpG dinucleotides can also stimulate a host immune response to the exogenous DNA or biological. Reducing the CpG dinucleotide content of codons allows the virus to avoid stimulating the host immune system as far as possible, which is beneficial to the hosts *in vivo* survival [52]. A low frequency of the CpG dinucleotides also affects the viral codon usage pattern.

### Correspondence analysis

Software to perform CA is available online at additional strategy for codon usage (http://mobyle.pasteur.fr/cgi-bin/portal.py?#forms::CodonW). This study also used an bias for MERS-CoV virus strains. Using the CA based on RSCU value, the patterns and trends of codon usage were observed, and the differences and evolution trends of the different strains were analysed. The effectiveness of this type of analysis was demonstrated by the known evolution of viral replication capacity, as well as revealing a new trend. The results also showed that the pattern of this CA could form a valuable tool for rapid classification and identification of any unusual patterns of newly isolated viruses. Using the RSCU values of the virus sequences of different CA groups provided an analysis and visualization of these data. For large multidimensional data sets, CA allows the reduction of the dimensionality of the data to effectively visualize and capture most of the changes that can occur [53].

### Principal component analysis

PCA is a common statistical method used to explain the codon usage of a specific gene. In the analysis, the RSCU value of each codon is explained by a 59-dimensional space and transformed into unrelated factors. In this model, PCA can determine any major variation from the RSCU value of each codon. Using both the PCA and correlation analysis, the factors influencing the codon usage bias can be determined effectively [13].

RSCU values of the 59 relevant codons were determined for all the sequences also studied in this work.

### Codon adaptation index

The codon CAI is one of the most widespread methods used to analyse codon usage bias resulting from natural selection pressure. In represents the adaption of the virus to the host. The CAI value ranged from 0 to 1. A higher CAI value indicates stronger adaption to the host. The codon usage patterns of the different host animals were obtained using an online tool (http://www.kazusa.or.jp/codon/). To estimate the codon adaption of the MERS to the host, the CAI value is calculated using the CAIcal software (http://genomes.urv.es/CAIcal). In the analysis, the synonymous codon usage pattern of the viral host were deposited as the reference and the CAI values of the MERS and MERS related strains were calculated after comparison with the reference from the different hosts.

### Hydropathicity and aromaticity indices

The hydropathicity and aromaticity of a single gene product are thought to be the result of translation selection resulting from natural selection [54]. Herein, the Gravy and Aroma score of each gene product were obtained using the Codon W program (version 1.4.2) to reflect the hydropathicity and aromaticity, respectively. A higher Gravy or Aroma score means that the protein is more hydrophobic or aromatic, respectively.

### Neutral evolution analysis

Neutral evolution analysis is used to estimate the varying role of mutational pressure and natural selection on the MERS and MERS related strains. In this analysis, the synonymous codon GC12s value was plotted against GC3s value [55]. To study the evolution characteristics of the mutation pressure and natural selection regression line in the MERS strains, the G12s or the G3s value was plotted against evolutionary time, respectively. The evolution speed resulting from the mutation pressure and the natural selection pressure was expressed as the slope of a simple regression line.

### Statistical analysis

Correlation analysis was performed using statistical software (version 20 and GraphPad Prism 6.0) for one-way analysis of variance (ANOVA), correlation analysis and to draw the figures. The 3D graph was created using Sigma Plot 12.5.

## SUPPLEMENTARY MATERIALS FIGURE AND TABLES




