# Supplementary material for: Analysis of the codon usage pattern in Middle East Respiratory Syndrome Coronavirus
Source: Oncotarget. 2017 Nov 27;8(66):110337–49. doi: 10.18632/oncotarget.22738 (PMC5746386; doi:10.18632/oncotarget.22738)
Supplement: Supplementary file 1 [file oncotarget-08-110337-s001.pdf]

Analysis of the codon usage pattern in Middle East Respiratory Syndrome Coronavirus

SUPPLEMENTARY MATERIALS

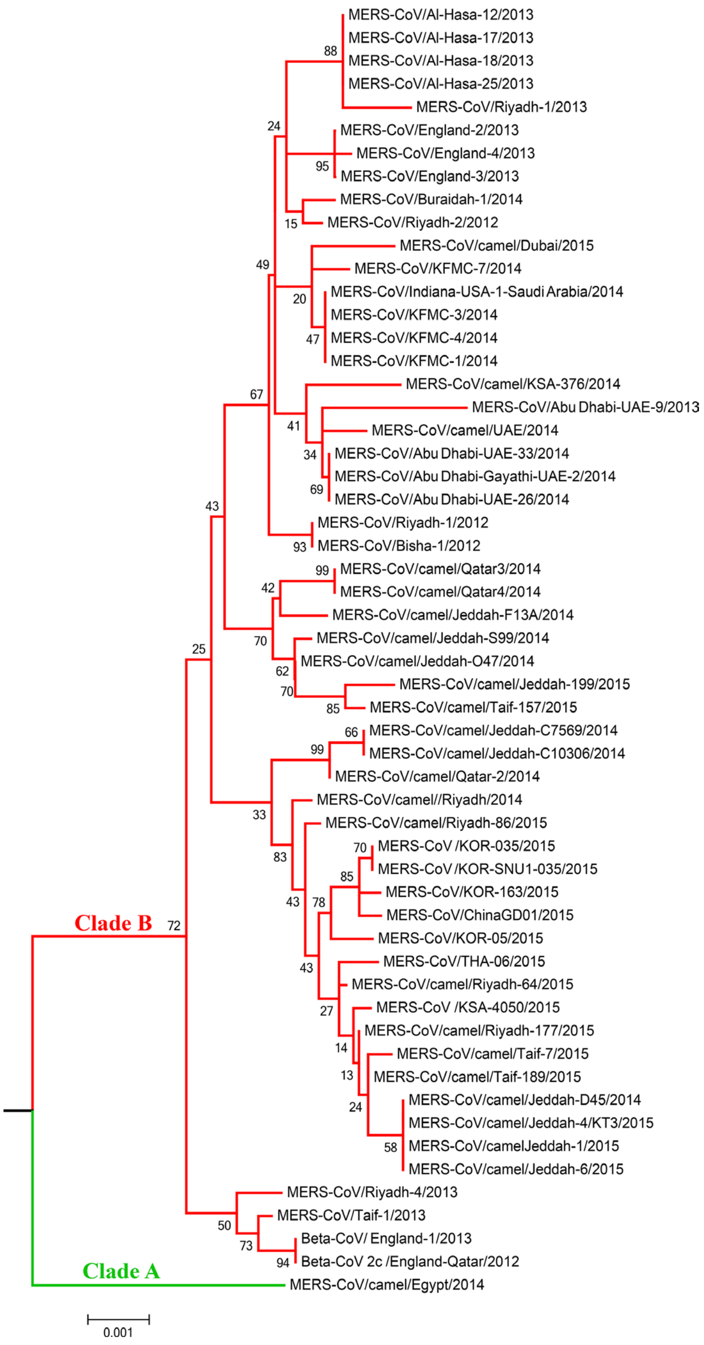

Supplementary Figure 1: Maximum-likelihood phylogenetic trees of 54 MERS-CoV and it be divided into two Clades. The green is belong to Clade A, and the red is belong to Clade B.

Supplementary Table 1: The detailed information describing the 71 Coronavirus strains used in our study

See Supplementary File 1

Supplementary Table 2: The mean and SD values of the nucleotide contents of different groups of MERS and MERS related isolates

		A%	C%	G%	T%
MERS isolates in human	Mean value	26.14	20.14	21.21	32.51
	SD value	0.02	0.05	0.10	0.05
MERS isolates in camel	Mean value	26.17	20.11	21.21	32.51
	SD value	0.03	0.08	0.07	0.03
MERS related isolates in bat	Mean value	26.86	19.41	21.32	32.43
	SD value	0.73	2.11	0.58	2.06
MERS related isolates in Erinaceus	Mean value	29.17	16.15	21.43	33.25
	SD value	0.01	0.02	4.35E-15	0.02

Supplementary Table 3: The synonymous codon usage pattern in human, camel, bat and hedgehog

See Supplementary File 1
