# Supplementary material for: Analysis of the codon usage pattern in Middle East Respiratory Syndrome Coronavirus
Source: Oncotarget. 2017 Nov 27;8(66):110337–49. doi: 10.18632/oncotarget.22738 (PMC5746386; doi:10.18632/oncotarget.22738)
Supplement: Supplementary file 2 [file oncotarget-08-110337-s002.doc]

**Supplementary Information**

**Table S1.** The detailed information describing the 71 Coronavirus strains used in our study

| No. | Strain Name | Accession Number | Year | Country |
| --- | --- | --- | --- | --- |
| 1 | Human betacoronavirus 2c England-Qatar/2012 | KC667074 | 2012 | England/Qatar |
| 2 | Betacoronavirus England 1 | KC164505 | 2013 | England |
| 3 | MERS CoV -Riyadh_1_2012 | KF600612 | 2012 | Saudi Arabia |
| 4 | MERS CoV -Riyadh_2_2012 | KF600652 | 2012 | Saudi Arabia |
| 5 | MERS CoV -Bisha_1_2012 | KF600620 | 2012 | Bisha |
| 6 | MERS CoV -England/4/2013 | KM210277 | 2013 | England |
| 7 | MERS CoV -England/3/2013 | KM210278 | 2013 | England |
| 8 | MERS CoV -England/2/2013 | KM015348 | 2013 | England |
| 9 | MERS CoV -Al-Hasa_18_2013 | KF600651 | 2013 | Al-Hasa |
| 10 | MERS CoV -Al-Hasa_17_2013 | KF600647 | 2013 | Al-Hasa |
| 11 | MERS CoV -Al-Hasa_12_2013 | KF600627 | 2013 | Al-Hasa |
| 12 | MERS CoV -Buraidah_1_2014 | KF600630 | 2014 | Buraidah |
| 13 | MERS CoV -Riyadh_4_2013 | KJ156952 | 2013 | Saudi Arabia |
| 14 | MERS CoV-Taif_1_2013 | KJ156949 | 2013 | Saudi Arabia |
| 15 | MERS CoV -Riyadh_9_2013 | KJ156869 | 2013 | Saudi Arabia |
| 16 | MERS CoV -Al-Hasa_25_2013 | KJ156866 | 2013 | Al-Hasa |
| 17 | MERS CoV -Abu Dhabi_UAE_9_2013 | KP209312 | 2013 | UAE |
| 18 | MERS CoV -Indiana/USA-1_Saudi Arabia_2014 | KJ813439 | 2014 | USA |
| 19 | MERS CoV -KFMC-7 | KT121581 | 2014 | Saudi Arabia |
| 20 | MERS CoV -KFMC-1 | KT121580 | 2014 | Saudi Arabia |
| 21 | MERS CoV -KFMC-3 | KT121573 | 2014 | Saudi Arabia |
| 22 | METS CoV -KFMC-4 | KT121575 | 2014 | Saudi Arabia |
| 23 | MERS CoV- Abu Dhabi_UAE_26_2014 | KP209313 | 2014 | UAE |
| 24 | MERS CoV-Abu Dhabi_UAE_33_2014 | KP209311 | 2014 | UAE |
| 25 | MERS- CoV Abu Dhabi/Gayathi_UAE_2_2014 | KP209310 | 2014 | UAE |
| 26 | MERS- CoV ChinaGD01 | KT006149 | 2015 | China |
| 27 | MERS-CoV/THA/CU/17_06_2015 | KT225476 | 2015 | Thailand |
| 28 | MERS-CoV/KOR/KNIH/002_05_2015 | KT029139 | 2015 | South Korea |
| 29 | MERS CoV -Korea/Seoul/SNU1-035/2015 | KU308549 | 2015 | Korea |
| 30 | MERS Cov -Hu/Riyadh_KSA_4050_2015 | KT026456 | 2015 | KSA |
| 31 | MERS CoV -KOREA/Seoul/035-1-2015 | KT374054 | 2015 | South Korea |
| 32 | MERS Cov -KOREA/Seoul/163-2-2015 | KT374050 | 2015 | South Korea |
| 33 | MERS CoV -Qatar4 | KF961222 | 2014 | Qatar |
| 34 | MERS CoV -Qatar3 | KF961221 | 2014 | Qatar |
| 35 | MERS -CoV | KJ477102 | 2014 | Egypt |
| 36 | MERS CoV -KSA-CAMEL-376 | KJ713299 | 2014 | KSA |
| 37 | MERS CoV -camel/Riyadh/Ry84N/2014 | KT368826 | 2014 | Saudi Arabia |
| 38 | MERS-CoV strain D2731.3/14 | KT751244 | 2015 | Dubai |
| 39 | MERS CoV -Camel/UAE/D1339.2/2014 | KP719931 | 2014 | UAE |
| 40 | MERS CoV -Camel/Qatar_2_2014 | KJ650098 | 2014 | Qatar |
| 41 | MERS CoV -Jeddah_C10306/KSA/2014-04-20 | KM027260 | 2014 | KSA |
| 42 | MERS CoV -Jeddah_C7569/KSA/2014-04-03 | KM027256 | 2014 | KSA |
| 43 | MERS CoV -camel/Jeddah/S99/2014 | KT368857 | 2014 | Jeddah |
| 44 | MERS CoV -camel/Jeddah/O47(b)/2014 | KT368852 | 2014 | Jeddah |
| 45 | MERS CoV -camel/Jeddah/D45/2014 | KT368837 | 2014 | Jeddah |
| 46 | MERS CoV -camel/Jeddah/F13A/2014 | KT368824 | 2014 | Jeddah |
| 47 | MERS CoV -camel/Taif/T157(b)/2015 | KT368890 | 2015 | Saudi Arabia |
| 48 | MERS -CoV-camel/Taif/T89/2015 | KT368885 | 2015 | Saudi Arabia |
| 49 | MERS CoV -camel/Taif/T7/2015 | KT368881 | 2015 | Saudi Arabia |
| 50 | MERS CoV -camel/Riyadh/Ry86/2015 | KT368879 | 2015 | Saudi Arabia |
| 51 | MERS CoV -camel/Riyadh/Ry177/2015 | KT368873 | 2015 | Saudi Arabia |
| 52 | MERS CoV -camel/Riyadh/Ry64/2015 | KT368877 | 2015 | Saudi Arabia |
| 53 | MERS CoV-camel/Jeddah/Jd199/2015 | KT368867 | 2015 | Jeddah |
| 54 | MERS CoV -camel/Jeddah/Jd6(b)/2015 | KT368860 | 2015 | Jeddah |
| 55 | MERS CoV-camel/Jeddah/Jd1(b)/2015 | KT368858 | 2015 | Jeddah |
| 56 | MERS CoV -camel/Jeddah/Jd4/2015 | KT368859 | 2015 | Jeddah |
| 57 | Bat coronavirus HKU5-1 | EF065509 | 2007 | Hong Kong |
| 58 | Bat coronavirus HKU5-2 | EF065510 | 2007 | Hong Kong |
| 59 | Bat coronavirus HKU5-3 | EF065511 | 2007 | Hong Kong |
| 60 | Bat coronavirus HKU5-5 | EF065512 | 2007 | Hong Kong |
| 61 | Bat coronavirus HKU5-1 | NC_009020 | 2016 | Hong Kong |
| 62 | Bat coronavirus HKU4-1 | EF065505 | 2007 | Hong Kong |
| 63 | Bat coronavirus HKU4-2 | EF065506 | 2007 | Hong Kong |
| 64 | Bat coronavirus HKU4-3 | EF065507 | 2007 | Hong Kong |
| 65 | Bat coronavirus HKU4-4 | EF065508 | 2007 | Hong Kong |
| 66 | BtTp-BetaCoV/GX2012 | KJ473822 | 2012 | China |
| 67 | Coronavirus Neoromicia/PML-PHE1/RSA/2011 | KC869678 | 2011 | South Africa |
| 68 | BtVs-BetaCoV/SC2013 | KJ473821 | 2013 | China |
| 69 | ErinaceusCoV/2012-216/GER/2012 | NC_022643 | 2012 | Germany |
| 70 | ErinaceusCoV/2012-216/GER/2012 | KC545386 | 2012 | Germany |
| 71 | ErinaceusCoV/2012-174/GER/2012 | KC545383 | 2012 | Germany |

The accession numbers marked in red, green, blue and black represent the MERS-CoV human isolates, the MERS-CoV camel isolates, the MERS-CoV related CoV bat isolates and the MERS-CoV related CoV hedgehog isolates, respectively.

**Table S3.** The synonymous codon usage pattern in human, camel, bat and hedgehog.

| AA | Codon | RSCU | | | | |
| --- | --- | --- | --- | --- | --- | --- |
|  |  | *Homo sapiens* | *Camelus*  *dromedarius* | *Taphozous* | *Pipistrellus*  *pipistrellus* | *Erinaceus*  *europaeus* |
| A | GCA | 0.910 | 0.660 | 1.164 | ***1.564*** | 0.680 |
|  | GCC | ***1.600*** | ***1.840*** | ***1.448*** | 0.696 | 1.110 |
|  | GCG | 0.430 | 0.400 | 0.324 | 0.520 | 0.240 |
|  | GCT | 1.060 | 1.070 | 1.060 | 1.216 | ***1.980*** |
| C | TGC | ***1.090*** | ***1.240*** | ***1.032*** | 0.500 | ***1.020*** |
|  | TGT | 0.910 | 0.760 | 0.968 | ***1.500*** | 0.980 |
| D | GAC | ***1.070*** | ***1.260*** | 0.932 | 0.870 | ***1.120*** |
|  | GAT | 0.930 | 0.740 | ***1.068*** | ***1.130*** | 0.880 |
| E | GAA | 0.850 | 0.840 | 0.966 | ***1.352*** | ***1.100*** |
|  | GAG | ***1.150*** | ***1.160*** | ***1.034*** | 0.648 | 0.900 |
| F | TTC | ***1.070*** | ***1.300*** | ***1.030*** | 0.814 | ***1.290*** |
|  | TTT | 0.930 | 0.700 | 0.970 | ***1.186*** | 0.710 |
| G | GGA | 1.000 | 0.850 | 1.284 | **2.048** | **1.290** |
|  | GGC | **1.350** | **1.640** | **1.372** | 0.976 | **1.290** |
|  | GGG | 1.000 | 0.930 | 0.628 | 0.684 | 0.830 |
|  | GGT | 0.650 | 0.580 | 0.716 | 0.292 | 0.600 |
| H | CAC | ***1.160*** | ***1.360*** | ***1.200*** | 0.782 | ***1.710*** |
|  | CAT | 0.840 | 0.640 | 0.800 | ***1.218*** | 0.290 |
| I | ATA | 0.510 | 0.330 | 0.630 | 0.651 | 0.420 |
|  | ATC | ***1.410*** | ***1.830*** | 1.098 | ***1.173*** | ***2.020*** |
|  | ATT | 1.080 | 0.840 | ***1.272*** | ***1.173*** | 0.550 |
| K | AAA | 0.870 | 0.840 | ***1.036*** | ***1.128*** | 0.910 |
|  | AAG | ***1.130*** | ***1.160*** | 0.964 | 0.872 | ***1.090*** |
| L | CTA | 0.430 | 0.550 | 1.026 | 0.570 | 0.560 |
|  | CTC | 1.170 | 1.330 | 1.002 | 1.332 | 1.130 |
|  | CTG | ***2.370*** | ***2.610*** | ***1.530*** | ***1.902*** | ***2.050*** |
|  | CTT | 0.790 | 0.660 | 0.930 | 0.666 | 1.130 |
|  | TTA | 0.460 | 0.300 | 0.870 | 0.666 | 0.280 |
|  | TTG | 0.770 | 0.540 | 0.648 | 0.858 | 0.850 |
| M | ATG | 1.000 | 1.000 | 1.000 | 1.000 | 1.000 |
| N | AAC | ***1.060*** | ***1.320*** | 0.946 | 0.816 | ***1.490*** |
|  | AAT | 0.940 | 0.680 | ***1.054*** | ***1.184*** | 0.510 |
| P | CCA | 1.100 | 0.980 | 1.188 | ***1.820*** | 1.210 |
|  | CCC | ***1.290*** | ***1.450*** | 1.136 | 0.608 | 1.170 |
|  | CCG | 0.450 | 0.510 | 0.316 | 0.120 | 0.340 |
|  | CCT | 1.140 | 1.060 | ***1.364*** | 1.456 | ***1.330*** |
| Q | CAA | 0.530 | 0.550 | 0.588 | ***1.242*** | 0.750 |
|  | CAG | ***1.470*** | ***1.450*** | ***1.412*** | 0.758 | ***1.250*** |
| R | AGA | ***1.290*** | 1.310 | 1.386 | ***2.544*** | 0.998 |
|  | AGG | 1.270 | ***1.330*** | 0.774 | 1.638 | ***1.839*** |
|  | CGA | 0.660 | 0.870 | ***1.590*** | 0.180 | 0.751 |
|  | CGC | 1.100 | 0.990 | 0.696 | 0.546 | 0.919 |
|  | CGG | 1.210 | 1.100 | 0.816 | 0.912 | 0.660 |
|  | CGT | 0.480 | 0.400 | 0.732 | 0.180 | 0.830 |
| S | AGC | ***1.440*** | 1.510 | 1.380 | 1.050 | 1.040 |
|  | AGT | 0.896 | 0.660 | 0.678 | 1.476 | 0.920 |
|  | TCA | 0.924 | 0.660 | 1.032 | ***1.788*** | 0.920 |
|  | TCC | 1.310 | ***1.840*** | ***1.494*** | 0.528 | ***1.530*** |
|  | TCG | 0.330 | 0.320 | 0.390 | 0.318 | 0.180 |
|  | TCT | 1.130 | 1.020 | 1.032 | 0.840 | 1.410 |
| T | ACA | 1.140 | 0.940 | 1.252 | ***1.432*** | 1.290 |
|  | ACC | ***1.420*** | ***1.790*** | ***1.356*** | 1.132 | ***1.380*** |
|  | ACG | 0.460 | 0.340 | 0.224 | 0.228 | 0.520 |
|  | ACT | 0.980 | 0.930 | 1.168 | 1.208 | 0.810 |
| V | GTA | 0.470 | 0.350 | 0.700 | 0.392 | 0.730 |
|  | GTC | 0.950 | 1.160 | 0.956 | 0.976 | 1.370 |
|  | GTG | ***1.850*** | ***1.930*** | ***1.556*** | ***1.756*** | ***1.490*** |
|  | GTT | 0.720 | 0.570 | 0.784 | 0.880 | 0.410 |
| W | TGG | 1.000 | 1.000 | 1.000 | 1.000 | 1.000 |
| Y | TAC | ***1.110*** | ***1.280*** | 1.000 | ***1.038*** | ***1.360*** |
|  | TAT | 0.890 | 0.720 | 1.000 | 0.962 | 0.640 |
| * | TAA | 0.880 | 0.690 | 0.213 | 0.000 | 0.000 |
|  | TAG | 0.710 | 0.380 | 0.144 | ***3.000*** | 1.500 |
|  | TGA | ***1.410*** | ***1.930*** | ***2.643*** | 0.000 | 1.500 |

The preferentially used codons for each amino acid are displayed in bold and italics. An asterisk (*) indicates a stop codon.
